# Short-Term Calorie Restriction in Male Mice Feminizes Gene Expression and Alters Key Regulators of Conserved Aging Regulatory Pathways

**DOI:** 10.1371/journal.pone.0005242

**Published:** 2009-04-16

**Authors:** Preston Wayne Estep, Jason B. Warner, Martha L. Bulyk

**Affiliations:** 1 Longenity, Inc., Lincoln, Massachusetts, United States of America; 2 Division of Genetics, Department of Medicine, Brigham and Women's Hospital and Harvard Medical School, Boston, Massachusetts, United States of America; 3 Department of Pathology, Brigham and Women's Hospital and Harvard Medical School, Boston, Massachusetts, United States of America; 4 Harvard-MIT Division of Health Sciences and Technology (HST), Harvard Medical School, Boston, Massachusetts, United States of America; Temasek Life Sciences Laboratory, Singapore

## Abstract

**Background:**

Calorie restriction (CR) is the only intervention known to extend lifespan in a wide range of organisms, including mammals. However, the mechanisms by which it regulates mammalian aging remain largely unknown, and the involvement of the TOR and sirtuin pathways (which regulate aging in simpler organisms) remain controversial. Additionally, females of most mammals appear to live longer than males within species; and, although it remains unclear whether this holds true for mice, the relationship between sex-biased and CR-induced gene expression remains largely unexplored.

**Methodology/Principal Findings:**

We generated microarray gene expression data from livers of male mice fed high calorie or CR diets, and we find that CR significantly changes the expression of over 3,000 genes, many between 10- and 50-fold. We compare our data to the GenAge database of known aging-related genes and to prior microarray expression data of genes expressed differently between male and female mice. CR generally feminizes gene expression and many of the most significantly changed individual genes are involved in aging, hormone signaling, and p53-associated regulation of the cell cycle and apoptosis. Among the genes showing the largest and most statistically significant CR-induced expression differences are *Ddit4*, a key regulator of the TOR pathway, and *Nnmt*, a regulator of lifespan linked to the sirtuin pathway. Using western analysis we confirmed post-translational inhibition of the TOR pathway.

**Conclusions:**

Our data show that CR induces widespread gene expression changes and acts through highly evolutionarily conserved pathways, from microorganisms to mammals, and that its life-extension effects might arise partly from a shift toward a gene expression profile more typical of females.

## Introduction

CR reproducibly extends the maximum and average lifespans of many different species, including some mammals [1] (for review see [2]). Increased lifespan also has been achieved in many of the same organisms through mutation of single genes, including key effectors of the interrelated Insulin/Insulin-like growth factor 1 (Igf1)/Growth hormone (Gh) signaling pathways [2], [3]. In the past few years cellular signaling pathways that interact with these hormonal pathways have yielded many mutants that extend lifespan in non-mammals. For example, many lifespan-extending mutations in the Akt/TOR (TOR, Target of Rapamycin, also known as Frap1 in mice and other mammals) nutrient-sensing pathway have been isolated in *Caenorhabditis elegans*, *Drosophila melanogaster*, and *Saccharomyces cerevisiae* [4]–[8].

TOR is a kinase that phosphorylates Akt1 [9], and it positively regulates translation by phosphorylation of at least two substrates: ribosomal S6 kinase (S6K) and 4E-BP1 (Eif4ebp1), a key translational repressor protein (for review see [10]). To date, no lifespan experiment in mammals has demonstrated the clear involvement of members of the TOR pathway even though this pathway is clearly involved in nutrient sensing and metabolic regulation, and likely plays a role in the response to CR. The sirtuin family of proteins also regulate some aspects of aging and it has been postulated that the life-extension benefits of CR are regulated through either the TOR or sirtuin pathways, or possibly both [11], [12]. However, the roles of these pathways remain in dispute [12].

Many previous aging and CR-related microarray studies have focused on gene expression changes in liver because it is a primary regulator of systemic metabolism, and of food energy processing, storage, and transport, and because it secretes and regulates the majority of circulating Igf1 [13]. Some studies have shown changes in transcript levels of key regulatory members of the Gh/Igf1 pathway [14], [15] and have even begun to connect the hormonal mechanisms governing the effects of CR in mammals to the cellular mechanisms found in other model organisms, e.g. the TOR pathway [16], [17]. Spindler and colleagues showed that mice begin to show persistent CR-dependent effects on liver gene expression as early as two weeks after initiation of CR, and that a majority of transcript changes at two weeks persist in long-term CR [18], [19]. This suggests short-term CR experiments might be useful in connecting the underlying mechanisms of CR in mice to those in other model organisms. However, these animals were fed only three times a week and fasted for at least 24 hours [19] and up to 48 hours prior to sacrifice [18]. This potentially masks important transcript changes that routinely occur differently in fully-fed versus CR animals, and Bauer and colleagues have shown that starvation of these durations results in even more and larger gene expression changes than were found in the CR studies of Spindler and colleagues [20].

In addition to CR there is another phenotype that confers greater longevity among many organisms, including most mammals: female gender [21], [22]. While it remains unclear whether or not female mice live longer in general than males, lifespans of both male and female mice are extended by CR and it is quite clear that their lifespans are greatly influenced by sex-associated variables, including hormonal status and reproductive history [23], [24]. It has been demonstrated that the gene expression differences between CR and control mice are similar to the differences found between long-lived dwarf mice and control mice, and also similar to female mice relative to male mice [25]. However, the shortest duration CR study included in this overall analysis consisted of 2 months of CR, the size of each of the CR and gender comparison groups was small (n = 4 and n = 6, respectively), and certain of these experimental designs included a period of starvation prior to sacrifice. To eliminate possible artifacts induced by starvation, Selman and colleagues employed a 16-day step-down CR protocol that avoided pre-sacrifice starvation [16]. Shorter duration studies like those conducted by Spindler and colleagues and Selman *et al.* have advantages over longer studies, in allowing much more rapid progress on the investigation of experimental regimens and compounds.

Here, we report the results of a two-week CR regimen designed to investigate the potential of such short-term studies to influence key gene expression changes. Importantly, prior microarray and metabolomic studies have provided many new insights into possible life-extension mechanisms of CR, but none has provided a systematic analysis of expression changes of genes known or suspected to be involved in aging-related pathways. In this study, we address these and related issues using liver microarray data generated from mice fed either high-calorie diets or short-term (14 days) CR diets absent prolonged starvation. Our results suggest that CR acts at least partly through highly evolutionarily conserved pathways, from microorganisms to mammals, and that the lifespan-extending effects of CR appear to overlap and might result from the lifespan-extending effects of femaleness.

## Results

### Dietary regimen dataset generation and statistical testing

To better understand the changes caused by CR, mice were fed diets high (“HIGHCAL”) or low (“CR”) in calories for 14 days and total liver RNA was used to produce expression profiles using spotted oligonucleotide DNA microarrays. Food was administered twice daily to reduce the complications of inducing a starvation response, including prior to sacrifice. Body weights of most mice stabilized after the first week and even the weights of most CR mice reached plateau by 7 to 9 days after initiation of CR. After background threshold adjustment and normalization our microarrays detected 8,347 non-redundant genes. To statistically test which genes are associated with CR relative to high calorie feeding, we used Significance Analysis of Microarrays (SAM) [26]. SAM performs a moderated t-test on two user-selected groups of microarray datasets, and returns a set of discriminant genes ranked in descending order by *d*, the t-statistic, and calculates a false discovery rate (FDR) based on random permutations of the data labels (see Methods). SAM also reports the *q*-value for each ordered gene (the lowest FDR at which the gene is called significant) and several other measures including fold-change values for each gene.

**Figure 1:**
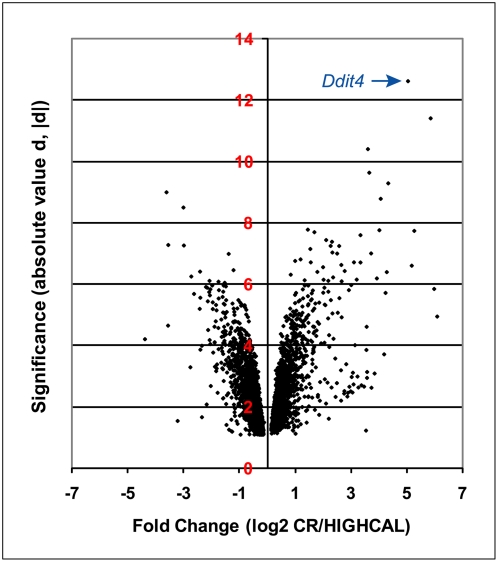
Plot of significance versus fold change of all significant genes. Plot of significance (absolute value of d, |d|) versus log2 fold change, for all 3,855 genes called significant at q<0.1. *Ddit4*, the gene with the highest significance score, is shown at upper right.

At a restrictive cutoff of q<0.01, 1,897 of 8,347 detected genes (22.7%) differed between CR and HIGHCAL (3,855 genes (46.2%) at the permissive cutoff of q<0.1). As shown in **Table S1**, Quantitative RT-PCR (Q-RT-PCR) confirmed direction of change (up or down) of 18 transcripts called by SAM as differentially expressed, and fold change values in most cases were larger for Q-RT-PCR (subsequent fold changes given are microarray values). The 90^th^ percentile number of median false positives at the permissive cutoff is about 634 genes. This suggests that even without considering the false negative rate the number of true positive genes changed significantly by CR can be conservatively estimated to be over 3,000. All genes called as significant at both cutoffs are listed in **Table S2**. Of the 1,897 genes changed by CR at q<0.01, 827 (43.6%) are upregulated and 1,070 (56.4%) are downregulated relative to HIGHCAL (at q<0.1, 1,693 (43.9%) are upregulated and 2,162 (56.1%) are downregulated). **Figure 1** shows all 3,855 genes in the permissive list (cutoff q<0.1) in a plot of significance (absolute value of *d*, |*d*|) versus log_2_ fold change. The shape of this plot shows that even though the majority of genes are downregulated, many upregulated genes show greater fold-changes and |*d*| scores than downregulated genes. Overall, 27 genes were upregulated more than 10-fold, while only 4 genes were downregulated more than 10-fold. This general asymmetry held true irrespective of the normalization method used.

### CR alters expression of key regulatory genes in conserved aging-related pathways

As highlighted in **Figure 1**, the single most significant and one of the most fold-changed discriminant genes is DNA-damage inducible transcript 4 (*Ddit4*), a p53-regulated negative regulator of the Frap1 pathway [27], which is increased by CR by over 33-fold (80.1-fold as measured by Q-RT-PCR). In addition, brief visual inspection of the significance-ranked list of genes reveals that many other top-ranked genes are involved in a variety of processes related to aging, hormonal synthesis and signaling, cell cycle regulation and apoptosis. To begin to further analyze these classes of significant genes in a systematic way we determined the intersection of these genes with the human and mouse genes listed in the GenAge database [28]. As described below, analyses for enriched Gene Ontology (GO) terms confirmed many of the broader general categories observed by visual inspection, however, GO terms and even GenAge annotations currently are too coarse to capture certain aging-related classes of genes and processes. One important excluded class among GO terms is genes that are known members of aging-related pathways but which have not been isolated as mutants that alter lifespan; key regulators of these pathways are of particular importance. The GenAge database includes many such genes. **Table 1** lists the intersection of our set of 1,897 significant genes (q<0.01) with the set of 265 non-redundant mouse and human genes listed in GenAge plus seven selected genes of this type that are not listed in GenAge but are among the most significant differentially expressed genes in our data.

**Table 1:** Aging-related genes upregulated or downregulated by CR, q<0.01.

Gene	Gene name	Fold Change	FC Rank	Stat Rank	Function
Ddit4*	DNA-damage-inducible transcript 4	*33.3*	*6*	*1*	Frap1 (mTOR) inhibitory tumor suppressor
***Lepr***	Leptin receptor	*58.4*	*3*	*2*	Regulator of adiposity and metabolism
***Aptx***	Aprataxin	*−2.3*	*139*	*6*	DNA repair, binds Parp1, Xrcc1, Xrcc4, p53
Cebpd*	CCAAT/enhancer binding protein delta	*4.3*	*74*	*12*	Mouse paralog swap increases lifespan
Ctsl*	Cathepsin L	*4.9*	*66*	*14*	Activates Plau by proteolytic cleavage
***Cdkn1a***	Cyclin-dependent kinase inhibitor 1A (P21)	*10.2*	*26*	*24*	Key negative regulator of cell cycle
Nnmt*	Nicotinamide N-methyltransferase	*36.5*	*5*	*26*	Yeast ortholog NNT1 mediates response to CR
Ppargc1a*	Ppargc1a, aka Pgc1-alpha	*6.0*	*52*	*55*	Sirt1-interacting regulator of metabolism
***Ctgf***	Connective tissue growth factor	*3.3*	*97*	*64*	Vascular growth factor; role in aging speculative
***Cebpb***	CCAAT/enhancer binding protein beta	*2.2*	*212*	*82*	Replacing *Cebpa* with *Cebpb* extends lifespan
***Tert***	Telomerase reverse transcriptase	*−2.9*	*76*	*88*	Telomerase reverse transcriptase
***Plau***	Plasminogen activator, urokinase	*2.7*	*144*	*92*	Overexpression in mice extends lifespan
Sgk1*	Serum/glucocorticoid regulated kinase	*4.6*	*72*	*94*	*C. elegans* ortholog mutation increases lifespan
***Map3k5***	Mitogen activated protein kinase kinase kinase 5	*2.0*	*274*	*107*	Involved in stress response and apoptosis
***Ar***	Androgen receptor	*−2.8*	*79*	*132*	Androgen receptor; dihydrotestosterone receptor
Igfals*	Insulin-like growth factor binding protein ALS	*−2.4*	*116*	*133*	Regulator of Igf1 signaling
***Blm***	Bloom syndrome homolog (human)	*4.7*	*67*	*186*	Involved in DNA repair; Wrn paralog
***Ghr***	Growth hormone receptor	*−2.1*	*186*	*190*	Mutation increases mouse lifespan
***Flt1***	FMS-like tyrosine kinase 1	*1.4*	*658*	*194*	Receptor for Vegf
***Foxo1***	Forkhead box O1	*1.5*	*540*	*228*	Ortholog of lifespan-extending *C. elegans* daf16
***Nr3c1***	Nuclear receptor subfamily 3	*−1.5*	*647*	*233*	Glucocorticoid receptor
***Stat3***	Signal transducer and activator of transcription 3	*1.7*	*414*	*233*	Signal transducer and activator of transcription 3
***Tnfrsf6***	Tumor necrosis factor receptor superfamily	*−1.5*	*682*	*244*	*Fas*; roles in apoptosis, development and cancer
***Jak2***	Janus kinase 2	*−1.4*	*854*	*246*	Associated with many genes linked to aging
***Ercc3***	*Ercc3*	*−1.3*	*1079*	*265*	Involved in DNA repair
***Gsta4***	Glutathione S-transferase	*−1.7*	*393*	*271*	Involved in oxidative protection
***Gclc***	Glutamate-cysteine ligase	*−2.0*	*230*	*316*	Overexpression in fruitflies extends lifespan
***Jun***	Jun oncogene	*2.1*	*237*	*328*	Pathway regulates aging in fruitflies and worms
***Nfkbia***	Nfkb inhibitor	*1.5*	*513*	*339*	Inhibitor of NF-kappa-B
***Txn1***	Thioredoxin 1	*−1.5*	*716*	*350*	Overexpression in mice extends lifespan
***Sncg***	Synuclein	*−2.0*	*237*	*354*	Synuclein, gamma
***Mapk9***	Mitogen activated protein kinase 9	*−1.3*	*1142*	*395*	Regulator of apoptosis and stress response
***Ppara***	Peroxisome proliferator activated receptor alpha	*−2.3*	*131*	*414*	Regulates fatty acid metabolism
***Foxo3***	Forkhead box O3	*1.5*	*511*	*421*	Orthologs regulate aging in fruitflies and worms
***Egfr***	Epidermal growth factor receptor	*1.7*	*420*	*432*	Regulator of cellular proliferation
***Top2a***	Topoisomerase (DNA) II alpha	*−1.5*	*596*	*465*	Topoisomerase indirectly linked to Wrn and Atm
***Hspa8***	Heat shock protein 8	*1.6*	*467*	*466*	Heat shock 70 kDa protein 8
***Tfdp1***	Transcription factor Dp 1	*−1.3*	*1203*	*527*	Involved in cell senescence, cell cycle, apoptosis
***Lmnb1***	Lamin B1	*−1.5*	*644*	*593*	Part of nuclear lamina, interacts with Lmna1
***Pdpk1***	3-phosphoinositide dependent protein kinase-1	*1.3*	*1114*	*594*	Phosphorylates and activates AKT1
***Cat***	Catalase	*−2.0*	*216*	*623*	Overexpression extends mouse lifespan
***Cebpa***	CCAAT/enhancer binding protein alpha	*−1.6*	*424*	*698*	Replacing *Cebpa* with *Cebpb* extends lifespan
***Vcp***	Valosin containing protein	*−1.3*	*1359*	*710*	ER protein/regulator of protein aggregation
***Gclm***	Glutamate-cysteine ligase	*−1.5*	*580*	*841*	Overexpression in fruitflies extends lifespan
***Pik3r1***	Phosphatidylinositol 3-kinase	*−1.4*	*799*	*857*	Involved in metabolism and insulin signaling
***Arhgap1***	Rho gtpase activating protein 1	*−1.3*	*1250*	*884*	Androgen receptor; dihydrotestosterone receptor
***Apex1***	Apurinic/apyrimidinic endonuclease 1	*−1.3*	*1357*	*1001*	Repairs oxidative DNA damage
***Xrcc5***	*Xrcc5*	*−1.3*	*1241*	*1017*	Involved in DNA repair /chromatin remodeling
***Mapk3***	Mitogen activated protein kinase 3	*−1.2*	*1683*	*1063*	Involved in stress response signaling

Genes are upregulated (positive fold-change values) or downregulated by CR, and are listed in the GenAge mouse and human database except for seven manually selected genes marked with an asterisk (*). Aging-related annotations come from GenAge [28] and other annotations come from Entrez Gene [73]. Fold change (FC) rank and statistical (Stat) ranks from SAM; separate statistical ranks are given for upregulated and downregulated genes.

Among aging-related genes upregulated by CR are leptin receptor (*Lepr*), cathepsin L (*Ctsl*), plasminogen activator urokinase (*Plau*), cyclin-dependent kinase inhibitor 1A (*Cdkn1a*) (a.k.a. *p21/Cip*), nicotinamide N-methyltransferase (*Nnmt*), peroxisome proliferative activated receptor, gamma, coactivator 1 alpha (*Ppargc1a*) (a.k.a. *Pgc-1 alpha*), serum/glucocorticoid regulated kinase 1 (*Sgk1*), and two *Cebp* paralogs: CCAAT/enhancer binding protein delta (*Cebpd*) and CCAAT/enhancer binding protein beta (*Cebpb*). Downregulated genes include aprataxin (*Aptx*), telomerase reverse transcriptase (*Tert*), insulin-like growth factor binding protein, acid labile subunit (*Igfals*), and growth hormone receptor (*Ghr*). *Lepr* was upregulated by over 58-fold by CR (49.3-fold by Q-RT-PCR) and was third in the list of genes ranked by significance. An increase in *Lepr* transcript abundance was somewhat expected since this is a reported result of CR [29] and of starvation [20] but the increase we observed was about 10-fold greater than seen in these previous reports. All of the genes in these lists are of possible importance in the life-extension response to CR but the large fold changes, high statistical ranks, and known roles in key aging-regulatory pathways make *Ddit4*, *Nnmt1* and *Cdkn1a* especially interesting.

### *4E-BP1* phosphorylation is reduced by CR

The large increase in expression of *Ddit4* suggests inhibition of the Frap1 (TOR) pathway by CR. Frap1 is a protein kinase whose currently known substrates include the kinases Akt1 and S6K1, and the translation regulatory protein Eif4ebp1 [9], [10]. Recent data suggest Frap1 enhances initiation of 5′-cap-dependent translation of most mRNA species by phosphorylation of Eif4ebp1, promoting its dissociation from the translation initiation factor Eif4e [30], [31]. Several sites on Eif4ebp1 are phosphorylated but phosphorylation of Thr69 (human Thr70) is a primary determinant of this dissociation [31]. Phosphorylation of S6K1 on Thr390 (human Thr389) is a primary activator of its kinase activity and appears to regulate translation of ribosome-related transcripts and ribosome biogenesis, although the mechanism underlying differential regulation of phosphorylation of Eif4ebp1 and S6K1 by Frap1 is currently unknown [31]. *Ddit4* has been shown to strongly inhibit Frap1 activity [32] and simply overexpressing *Ddit4* is sufficient for this inhibition [33]. Therefore, the over 33-fold increase of the *Ddit4* transcript in CR mice suggested a reduction of phosphorylated isoforms of substrates of Frap1 in livers of these mice.

**Figure 2:**
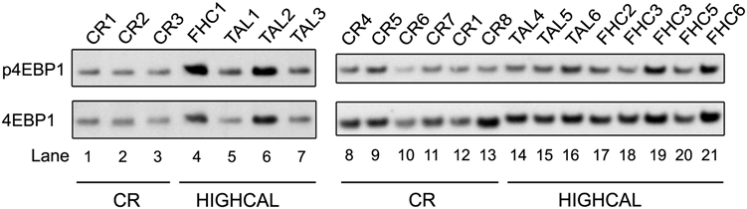
CR-induced phosphorylation changes in Eif4ebp1. Western blots of unphosphorylated Eif4ebp1 (4EBP1) and Thr69 phosphorylated Eif4ebp1 (p4EBP1) for calorie restricted (CR) and high-calorie-fed (HIGHCAL) mice. Labels at top indicate individual mouse sample codes. CR, Calorie Restriction; FHC, Fixed High Calorie feeding; TAL, True Ad Libitum feeding. TAL1, TAL3, and TAL4 are from livers used to make the RNA pool TAL-P. CR1, CR7, and CR8 are from livers used to make the RNA pool CR-P1. CR4, CR5, and CR6 are from livers used to make the RNA pool CR-P2. FHC3 and FHC3B are separate samplings of the same liver.

To test this hypothesis we performed Western blots on Eif4ebp1 and S6K. Two primary isoforms of S6K1, p70 and p90, were detected in our liver samples. We assayed Thr390 of p70 (Thr412 of p90), and for Eif4ebp1 we assayed Thr69, which are recognized targets of phosphorylation by Frap1. Consistent with the current model of the regulation of Eif4ebp1 by Frap1, Thr69 phosphorylation of Eif4ebp1 was increased more than 2-fold by high calorie feeding relative to CR (**Figure 2**) (*P* = 6.0×10^−4^, one-sided Wilcoxon rank sum test). In contrast, the Thr390 phosphorylated p70 isoform of S6k1 was undetectable in our liver samples, including those from TAL-fed mice, while Thr412 of p90 was detected but no significant change was found in HIGHCAL relative to CR mice. These results suggest that CR results in reduced efficiency of initiation of cap-dependent translation through phosphorylation of 4EBP1 but does not significantly alter processes dependent upon phosphorylation of S6K1, such as translation of ribosomal transcripts and ribosome biogenesis [34].

### CR alters gene expression within specific gene classes

To further understand and quantify enriched classes of genes significantly upregulated or downregulated by CR we employed the GO analysis tool FuncAssociate [35], which returns all overrepresented GO terms for a given set of input genes. We separately analyzed upregulated, downregulated, and the combined upregulated and downregulated sets of genes using both permissive and restrictive cutoffs for a total of 6 separate analyses. The number of terms returned for each of the upregulated (U), downregulated (D), and combined upregulated and downregulated (C) sets of genes are as follows: at the restrictive cutoff q<0.01 U = 57, D = 61, and C = 103; at the permissive cutoff q<0.1 U = 94, D = 99, and C = 170. All GO terms and statistics can be seen in **Table S3**.

**Figure 3:**
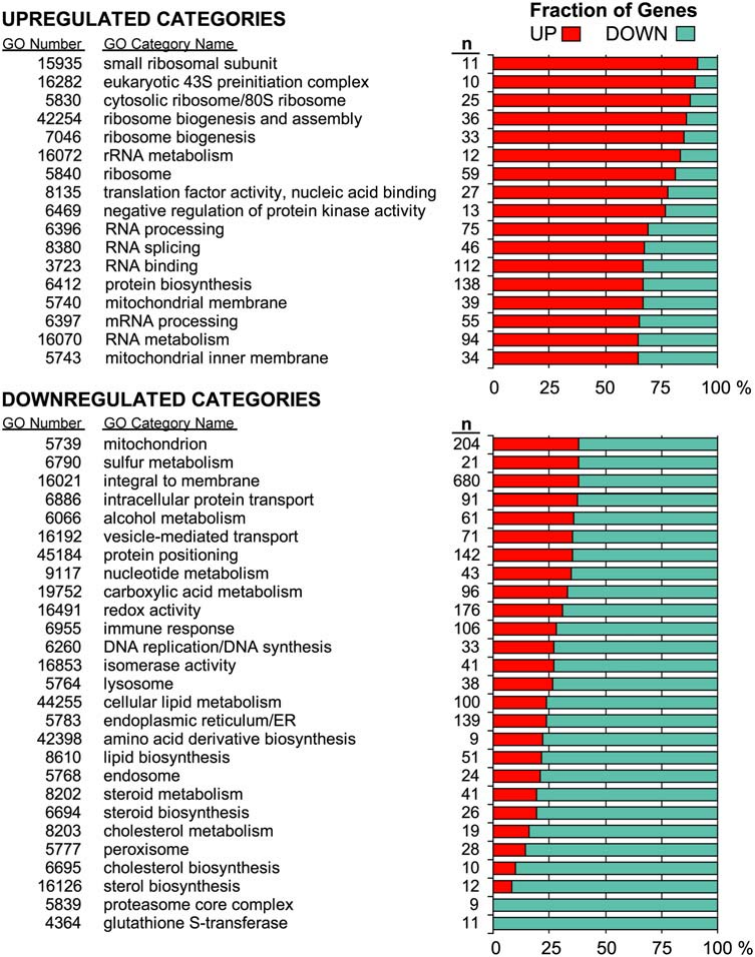
GO categories with largest relative fractions of upregulated or downregulated genes. Each stacked bar graph displays the relative upregulated or downregulated fractions of the total number of genes (n = 100%) significantly altered by CR within a given GO category returned by FuncAssociate (adjusted p<0.05). The upregulated fraction is shown in red and the downregulated fraction is shown in blue.

To more clearly understand the changes in biological function that result from changes in gene expression, for each GO term we plotted in a stacked bar graph the relative fraction of upregulated and downregulated genes associated with that GO term from the SAM output (q<0.1). **Figure 3** shows a selection (culled to eliminate excessive redundancy) of the GO terms with the largest fractions of upregulated or downregulated genes. CR results in a clear relative upregulation of genes involved in protein biosynthesis, rRNA processing, mRNA metabolism and splicing, ribosome, and regulation of protein translation. Many GO categories consist mostly of downregulated genes including those involved in mitochondria, carboxylic acid metabolism, DNA replication, steroid, cholesterol and lipid metabolism, lysosome, peroxisome, and glutathione S-transferase activity. Several downregulated GO classes are involved in protein turnover including endoplasmic reticulum/ER, isomerase activity, amino acid derivative biosynthesis, protein positioning, and the proteasome.

### CR feminizes gene expression overall and within specific gene classes

Since genes involved in hormone biosynthesis are generally downregulated in our data and several genes previously demonstrated to display sexually dimorphic expression are found at the top of our statistical and fold-change rankings, we suspected CR might have an overall feminizing effect on gene expression. To test this hypothesis we compared our data to the male versus female liver expression microarray data of Yang and colleagues which were collected using 334 total microarrays (one microarray per liver sample; 165 male and 169 female mice) [36]. Of the 1,897 genes in our restrictive list, 872 were found in their study to be more highly expressed in either male or female (1,711 of 3,855 genes in our permissive list).

**Figure 4:**
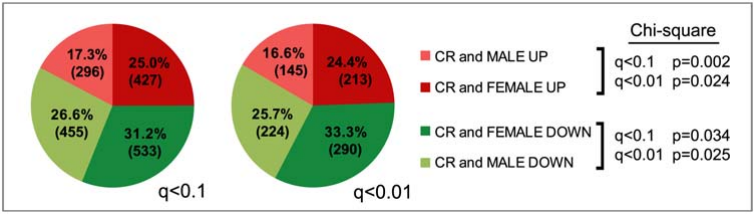
CR feminizes overall gene expression. Fractional distributions of all genes changed by CR and which display sexually dimorphic expression in the data of Yang et al. Percent numbers are percent of all genes displaying both specific directional changes from CR and sexually dimorphic expression and corresponding numbers of genes in each of these four classes are given in parentheses. Deviation from expected frequencies was determined by a chi-square test for upregulated genes and separately for downregulated genes, at each q-value cutoff.

**Figure 4** displays the frequencies (at both permissive and restrictive cutoffs) of each of four classes of genes: increased by CR and more highly expressed (UP) in females, increased by CR and UP in males, decreased by CR and displaying lower expression (DOWN) in females, and decreased by CR and DOWN in males. The percentage of genes displaying feminized expression is higher among the set of genes upregulated in CR than among genes downregulated in CR. Separate Chi-square analyses of CR-upregulated genes and CR-downregulated genes at both the restrictive and permissive cutoffs show a significant bias toward a female pattern of expression. We also used FuncAssociate to determine the overrepresented GO category associations of each of these four gene sets. As shown in **Table 2**, genes downregulated and masculinized by CR are involved in immune response; genes upregulated and feminized by CR are involved in general metabolism and protein biosynthesis; and genes downregulated and feminized by CR are involved in enzymatic catalysis, and protein modification and transport through the Golgi and endoplasmic reticulum. These results suggest that CR has a generally feminizing effect on gene expression, which involves protein metabolism and turnover, which might contribute to the lifespan extension effects of CR.

**Table 2:** GO categories associated with groups of genes displaying CR-regulated and sex-biased expression.

Genes UPREGULATED in CR			Genes DOWNREGULATED in CR		
***CR and Female UP***			***CR and Female DOWN***		
**Overrepresented GO Attributes**			**Overrepresented GO Attributes**		
**P-adj**	**Number**	**GO Attribute**	**P-adj**	**Number**	**GO Attribute**
<0.001	31	0005840: ribosome	0.001	42	0005783: endoplasmic reticulum
<0.001	64	0009058: biosynthesis	0.023	32	0005794: Golgi apparatus
<0.001	217	0008152: metabolism	0.031	199	0003824: catalytic activity
***CR and Male UP***			***CR and Male DOWN***		
**Overrepresented GO Attributes**			**Overrepresented GO Attributes**		
**P-adj**	**Number**	**GO Attribute**	**P-adj**	**Number**	**GO Attribute**
-	-	NONE	<0.001	34	0006952: defense response

Columns display GO attribute categories associated with genes upregulated or downregulated in CR (q<0.1) and also relatively upregulated in either female or male sex according to the data of Yang *et al.* [36]. Adjusted p-values and number of genes are given for each category.

## Discussion

A primary focus of biomarker and lifespan studies has been calorie restriction (CR). Spindler and colleagues first showed that two weeks of CR (40% reduction relative to ad libitum) causes many changes in mouse gene expression that persist in the long term [19]. But the small number of increased or decreased transcripts found in their experiments, prolonged periods of starvation of animals including prior to sacrifice, and a report by Bauer and colleagues showing that a greater number of transcripts are changed by starvation alone highlighted the need for a strategy to minimize starvation [19], [20]. Selman and colleagues subsequently utilized a 16-day CR protocol that steps down from 90%, to 80%, and finally to 70% caloric intake (30% CR), and which avoids pre-sacrifice starvation. We employed a hybrid strategy that utilizes a CR treatment similar to that administered by Spindler and colleagues (approximately 40% CR for 2 weeks), but that avoids the induction of a starvation response. To enrich for chronic CR-related gene expression responses and to minimize the starvation response, we fed mice twice daily, including about half a day prior to sacrifice. This approach is more consistent with CR assays in organisms with constant food access, including yeast, nematodes, and fruitflies. Using this approach we found that CR alters liver expression of at least 3,000 genes. Comparisons of lists of genes most significantly associated with CR to those listed in GenAge allowed us to systematically discover important aging regulatory genes in our data, although current limitations of both GenAge and Gene Ontology annotations motivated a search for additional aging-related genes among the genes displaying the most significant expression differences between CR and high-calorie feeding.

*Nnmt*, *Cdkn1a*, and *Ddit4* are of particular interest because of their high statistical rankings, large fold-changes and because all three appear to play key roles in aging regulatory pathways. Other notable upregulated genes are *Lepr*, *Cebpd*, *Cebpb*, *Ctsl*, *Plau*, *Sgk1*, and *Ppargc1a* (*Pgc-1 alpha*), and notable downregulated genes include *Aptx*, *Tert*, *Igfals* and *Ghr*. Previous microarray studies of CR have shown changes in *Ddit4*, *Ghr* [29] and *Sgk1* [37] but not in most of these other genes. Bauer and colleagues reported the upregulation of leptin receptor (*Lepr*) with starvation, and in our data it is upregulated about 50-fold as measured by both microarray and Q-RT-PCR [20]. Leptin is closely associated with the neuroendocrine responses of starvation, CR, and body size [38]; however, the direct role of leptin in aging and its modulation by CR remain uncertain, but a primary regulatory role of leptin in the adaptive response of CR has been proposed [39]. Two pairs of these genes are notable because they are functionally related. *Ctsl* encodes a protease that cleaves Plau, and *Cebpd* and *Cebpb* are paralogs (they are also homologs of *Cebpa*, which appears farther down Table 1). Neither *Ctsl* nor *Cebpd* has been directly implicated in aging; however, our results suggest they might be involved in the lifespan regulation observed in mutants in the other member of these pairs.

Both *Ppargc1a* and *Nnmt* have been shown to function in sirtuin-related pathways. *Ppargc1a* is a multifunctional regulator of metabolism and mitochondrial biogenesis, and has been linked to regulation of glucose homeostasis, CR and a possible role in aging through a physical interaction with Sirt1 in liver [40]. *Nnmt* encodes Nicotinamide N-methyl transferase which produces N1-methyl nicotinamide from the vitamin B3 precursor nicotinamide. Nicotinamide partly or completely abrogates yeast lifespan extension by CR, possibly by increasing cellular levels of NADH, a competitive inhibitor of the Sir2 protein [41], [42]. Overexpression of human *NNMT* in yeast increases rDNA silencing, a phenomenon linked to sirtuin-mediated extension of life span [41]. Overexpression in yeast of *NNT1*, the yeast homolog of *Nnmt*, also increases rDNA silencing, increases lifespan which is not further increased by glucose restriction, and restores full lifespan extension by calorie restriction in a *pnc1* mutant [41], [42]. Tsuchiya and colleagues showed that elevated nicotinamide blocks CR-induced lifespan extension in yeast lacking sirtuins [43]. This suggests a Sir2-independent mechanism for abrogation of lifespan extension by nicotinamide. Taken together, these results suggest that *NNT1* overexpression in yeast recapitulates a Sir2-independent life extension mechanism governed by CR. Our data showing that its mammalian homolog, *Nnmt*, is among the most statistically significant and most upregulated genes (increased by approximately 48-fold as measured by Q-RT-PCR) in response to CR, strongly suggests that CR in mammals might be mediated by a similar and highly evolutionarily conserved mechanism.

Our results confirm those of others suggesting that regulation of DNA replication and repair, apoptosis, and cell proliferation play important roles in the CR response. Aprataxin (*Aptx*) is one of the most significant downregulated genes and is involved in the repair of multiple types of DNA lesions and interacts with the DNA repair and stress-response proteins Parp1, Xrcc1, Xrcc4, and p53. The p53-regulated key regulator of apoptosis and cell cycle progression *Cdkn1a* (increased by approximately 22-fold as measured by Q-RT-PCR) is among the most significant upregulated transcripts in our data and is regulated post-translationally by Akt phosphorylation [44], [45]. Telomerase reverse transcriptase (encoded by *Tert*, decreased by approximately 2.9-fold) is also a key regulator of cell proliferation and apoptosis, and has been found in a multiprotein complex with Akt, S6K and Frap1 (TOR) [46].

The Frap1 pathway genes *Ddit4* and *Sgk1* also have not previously been shown to be involved in regulating aging in mammals. However, it has been shown that *Ddit4* expression changes in response to CR in both rats and mice [16], [17], that the Akt/TOR pathway regulates lifespan in multiple species [4], [5], [7], [8], that *Sgk1* acts in this pathway to regulate nematode lifespan [6], and that *Ddit4* regulates this pathway in mammals [32], [47]. In *C. elegans* SGK-1 acts to mediate DAF-2/Igf1 signaling in a complex with Akt kinases and partial deletion or knockdown of *SGK-1* increases lifespan [6]. The expression of *Ddit4* is regulated by hypoxia, cellular energy stress, and by p53 and p63 in response to DNA damage and reactive oxygen species (ROS) [33], [47], [48]. *Ddit4* and its paralog *Ddit4l* are evolutionarily conserved and the fruitfly orthologs *Scylla* and *Charybdis* appear to function in a similar manner. Overexpression of *Scylla* and/or *Charybdis* results in smaller cell size and small flies, and loss of both genes results in larger cells and flies [49].

Recent data from Ellisen and colleagues suggest that a primary *Ddit4* mechanism of action is to competitively bind inhibitory 14-3-3 proteins that otherwise bind to the Tsc2 protein, part of the tuberous sclerosis complex that inhibits Akt/Frap1 signaling when Tsc2 is unbound [32]. Hypoxia or energy stress triggers the binding of 14-3-3 proteins by Ddit4, freeing Tsc2 to suppress Frap1 kinase activity and reduce phosphorylation of its substrates. These authors showed that mouse embryo fibroblasts (MEFs) deleted for *Ddit4* demonstrate increased proliferation and anchorage-independent growth resulting from dysregulation of Akt/Frap1 pathway signaling [32]. They further demonstrated that *Ddit4*-mediated inhibition of Frap1 occurs even in the presence of constitutive Akt activation. Subcutaneous injection into nude mice of MEFs containing constitutively activated Akt results in slow-growing tumors, and the additional deletion of *Ddit4* causes much more rapid tumor growth. These data suggest that *Ddit4* is a tumor suppressor acting on the Frap1 pathway downstream of Akt, and *Ddit4* downregulation has recently been described in a subset of human cancers [32].

Since overexpression of *Ddit4* in cultured mouse cells is sufficient to inhibit activation of the Frap1 pathway, greatly reducing phosphorylation of S6K1 and ribosomal protein S6 [33], the over 33-fold increase that CR elicits in *Ddit4* in our experiments suggested a strong CR-dependent downregulation of Frap1 signaling in liver. This was confirmed by Western blots of Thr69 of Eif4ebp1 [47], [50]. Sharp and Bartke also found decreased phosphorylation of Eif4ebp1 in livers of Ames (Prop1df) dwarf mice, but they also found decreased phosphorylation of the p70 and p85/p90 isoforms of ribosomal S6K [51]. Jiang and colleagues also reported decreased phosphorylation of Thr389 of liver p70S6K in rats undergoing 4-week CR, as well as decreases in overall Frap1 kinase activity and phosphorylation of critical residues on Eif4ebp1, Akt and Tsc2 [52]. We did not find S6K to be differentially phosphorylated between CR and HIGHCAL mice. This result needs to be investigated further since phosphorylated p70S6K, a primary phosphorylation target of Frap1, was not detectable in our samples, and it therefore appears that phosphorylation of p70 and p90 isoforms are differently regulated from one another, and from Eif4ebp1. Nevertheless, increased phosphorylation of Eif4ebp1 and the large increase in the *Ddit4* mRNA transcript together suggest CR results in inhibition of the Frap1 pathway in liver to inhibit translational initiation. It will be of interest to determine which of these observed post-translational changes persist in long-term CR. Dhahbi and colleagues have shown that certain genes display oscillatory expression over the initial weeks of CR [19]. Thus, it is possible or even likely that key post-translational modifications also oscillate, or that the 2-week CR protocol used in this and similar studies is too short to induce important post-translational and other phenotypic alterations that occur in long-term CR.

Our GO analysis shows that CR results in relative upregulation of genes within GO categories associated with ribosomes, translation initiation and elongation, and protein biosynthesis, suggesting that protein biosynthesis and processing machinery remain at fairly high levels. Our Western blot data suggest that translation is more tightly regulated through Frap1-dependent phosphorylation events. It is quite interesting that genes encoding ribosomal proteins and translation initiation and elongation factors are almost uniformly upregulated in response to CR, but translation appears to be downregulated. These findings are consistent with the possibility that many of the effects of CR are mediated to some degree through the control of protein synthesis and degradation. This possibility is supported by the findings that lifespan can be extended by mutations in translational regulatory proteins and in many individual ribosomal proteins in both yeast and *C. elegans* (see [53] for review), and less directly in more complex organisms by the observation that tryptophan restriction extends lifespan in rats [54] and methionine restriction extends lifespan in both rats and mice [55], [56]. Consistent with this model, downregulated GO categories in our data include several involved in protein turnover including endoplasmic reticulum/ER, isomerase activity, amino acid derivative biosynthesis, proteasome core complex, and protein positioning and transport.

Our GO analysis also shows that CR results in relative downregulation of genes within GO categories associated with peroxisomes, lysosomes, mitochondria, and metabolism of lipids, cholesterol, and steroid hormones. Our results are in some cases consistent with but also contrast the GO analyses reported by Selman and colleagues resulting from mice treated with acute CR for 16 days [16]. They also found that genes involved in lipid and steroid metabolism were upregulated but in contrast to our results they found a subset of lipid metabolism genes downregulated; they found the GO category for lipid metabolism (GO:0006629) among both upregulated and downregulated categories. Also in opposition to our findings, they found mitochondrion (GO:0005739) and membrane fraction (GO: 0005624) to be upregulated categories, and metabolism (GO:0008152) to be downregulated. However, we found a much larger number of differentially expressed genes in each category, which might provide an explanation for these discrepancies. For example, even at the restrictive cutoff used in our analysis there are 345 differentially expressed genes in the metabolism GO category compared to 181 in their data. These additional genes might be responsible for the disparate findings.

Our results also show a clear alteration in hormone biosynthesis and suggest that even short-term CR biases expression changes toward a more feminine profile. This hypothesis was confirmed by comparing our data to the whole-genome survey of sexually dimorphic gene expression performed by Yang and colleagues on 165 male and 169 female mice [36]. This confirms the findings made by Swindell in an analysis of longer duration (between 2 and 17 months) CR experiments on male mice, and sexually dimorphic expression on small groups of female and male mice (n = 6 in each group) [25]. Overall, our results showing downregulation of genes involved in sterol, steroid, and sex hormone production by CR suggest a possible primary or significant contributory mechanism for some of the hormonal changes in response to CR in several species, including humans. Even more importantly, these changes further tie CR to key hormonal and cellular effectors of aging in model organisms, and possibly even in humans, since in humans and most other mammals females live longer than males [21], [22].

However, even though CR extends lifespan in both male and female mice there are two primary barriers to establishing simple relationships between CR, gender, hormones, and lifespan, and extrapolating mouse and other rodent results to humans and other primates: 1) there is clear disagreement among prior reports regarding the effects of CR on various hormone levels in rodents, humans and other primates [57]–[60]; and, 2) there is disagreement among previous studies regarding gender effects on mouse lifespan. Reports of CR-induced changes of important hormone biomarkers such as GH, IGF1, and DHEA-S (dehydroepiandrosterone sulfate) are variable across and even within species. These variable results are counterbalanced somewhat by the fact that the effects of CR on insulin levels and the insulin axis appear to be mostly consistent across species [57], [60]. Insulin is a key longevity biomarker but the insulin axis is complex and is regulated by many other hormones, including sex and adrenocortical hormones [60].

Importantly, it remains unclear whether or not there are gender differences in mouse lifespan. One large and early study suggests females live longer than males, especially virgin females [24]. This study from the Jackson Labs remains among the largest, with data on thousands of mice, but later data from this same facility cast doubt on these prior results, as improved conditions allowed far greater lifespans [24]. More recent but still unpublished data from this facility on 3,744 mice of 32 different laboratory strains suggest that females might not live longer [61], supporting prior data presented by others including Ingram *et al.* [62]. Until final data from this and other high-quality studies are published, whether or not there is a difference between male and female mouse lifespan will remain an open question. Nevertheless, our data raise some intriguing questions including 1) are hormonal aspects or other determinants of gender also important regulators of lifespan; and 2) what might be the effect of CR on gene expression in female mice? It is possible that the response will be similar to what we found in male mice, i.e. many of the same genes will change in a trajectory away from male expression levels. It will be important to test this possibility and determine whether or not CR induces feminizing gene expression changes in organisms that have a clear female lifespan advantage, such as humans, and whether such changes might influence lifespan.

## Materials and Methods

### Animals and dietary regimens

Nine-month-old retired breeder ICR male mice were obtained from Harlan Breeders (Indianapolis, IN). Animals were kept in a positively pressurized HEPA air-filtered animal room with 12-hour light/dark cycles. A total of 35 mice were fed 3 specified dietary regimens. Six mice were fed a True Ad Libitum (TAL) diet of which they ate as much as they desired, 12 mice were fed calorie-restricted diets of 73 kcals per week (CR), and 17 mice were fed a diet of 110 kcals per week (Fixed High Calorie, FHC). Recovered food from TAL mice suggested an average consumption plus loss of 149 kcal/week, however, a small amount of the food clearly was lost in bedding, and a more accurate estimate is probably between 120 and 130 kcal/week. Therefore, we estimate the approximate percent calorie reduction for each fixed-calorie diet relative to TAL is 10% to 15% for FHC and 40% to 45% for CR. Calorie reduction of CR relative to FHC is 34%. All mice were given non-acidified water ad libitum throughout regimen treatment.

Mice were fed freshly hydrated food every twelve hours at the beginning and end of the light cycle. Purified diets differing in caloric composition but similar in macronutrient ratios to AIN-93M (Diet No. F05312, Bioserv, Frenchtown, NJ) were assembled from individual ingredients and vitamin and mineral mixes (Bio-Serv, Frenchtown, NJ). These diets consisted of the following approximate caloric composition: 10% fat, 15% protein, and 75% carbohydrate. Animals were fed 12 to 16 hours prior to sacrifice and remaining food was removed after feeding FHC and TAL mice. Animals were sacrificed by rapid decapitation. Tissues were immediately harvested and flash frozen in liquid nitrogen, placed in polypropylene tubes and placed in cryogenic storage for future processing. All animal housing and experiments were performed in compliance with prevailing local, state, and federal regulations of Waltham, MA, USA, and according to the guidelines of Suckow *et al.* [63] and the Institute for Laboratory Animal Research (ILAR) [64].

To assess the possibility of minimizing expenses over the long-term some RNA samples were combined into pools of 3 prior to cDNA synthesis and hybridization to single microarrays. Pooling of RNA samples for microarray analysis has been reported to reduce intrasample variance and results in averaging of most genes, but does not otherwise affect the analysis [65]. In our experiments all microarrays of pooled samples were analyzed identically with microarrays from individual samples. The 8 CR microarrays consist of 6 microarrays from individual samples and 2 microarrays each produced using pooled RNA samples from 3 other individual mice. The 15 high calorie microarrays consist of 3 individual and 1 pool of TAL samples and 8 individual and 3 pooled FHC samples.

### Microarray experiments

Spotted, long oligonucleotide microarrays representing ∼19,000 genes, duplicates and controls (17,752 non-redundant genes and ESTs) were used to interrogate mRNA abundance (GEO platform accession GPL6761; GEO Samples GSM283874 through GSM283896). The oligonucleotide library was designed by Compugen and manufactured by Sigma-Genosys. Liver samples were taken from cryogenic storage and fragmented in liquid nitrogen. Total RNA was purified by Qiagen RNeasy Mini kit. Briefly, several small pieces totaling 200 mg taken from different locations from an individual liver were homogenized in Qiagen guanidine isothiocyanate lysis buffer, and then subsequent steps were performed according to the Qiagen protocol. All pools of RNA were made by combining equal amounts of each individual RNA sample subsequent to purification and quantification of RNA samples from the individual livers. Microarray design and preparation, sample labeling, and microarray hybridization and scanning services were performed by the Harvard Partners Center for Genetics and Genomics (now Partners Center for Personalized Genetic Medicine). Stratagene Universal Mouse Reference RNA was chosen as a control for two-color hybridization [66]. Fluorescent labels were incorporated directly during cDNA synthesis using the ChipShot direct labeling and cleanup system according to the manufacturer's instructions (Promega, Madison, WI). Total liver RNA was labeled with Cy3 and control RNA was labeled with Cy5. Following dye incorporation and sample cleanup, sample cDNA and control cDNA were mixed together with spiked controls that aid in hybridization calibration, and then the mixed sample was hybridized to the microarray surface using a Maui Hybridization System (Biomicro, Salt Lake City, UT). Microarrays were washed and scanned at 532 nm and 635 nm wavelengths on an Axon 4000B microarray scanner (Axon Instruments, Union City, CA).

### Microarray data collection, processing, and analysis

Scanned data were collected and processed using GenePix 4.1 software (Axon Instruments, Union City, CA). Genes with median spot intensities below 50 for liver expression for both CR and HIGHCAL were culled from the data set to give a set of 9,550 genes and controls for normalization. These culled data were normalized using the CARMAweb implementation of the limma package for R. Median background was subtracted from median foreground signal using the normexp method followed by loess within-array normalization and then scale between-array normalization [67], [68]. To further eliminate genes near background in these normalized data we set a median expression threshold value of 90 for either CR or HIGHCAL. These adjustments gave a final normalized and culled set of 8,347 genes and controls for statistical testing. For testing of significance we used the software package, Significance Analysis of Microarrays (SAM) and implemented the two class unpaired option [26]. SAM computes and returns a ranked list of significant genes and several statistics, including the modified t-statistic *d*, and the median false discovery rate (FDR). The FDR increases with the number of genes called significant and is the median number of genes falsely called significant from all permutations divided by the number of genes called significant at a user-specified cutoff. The q-value is the lowest FDR at which a given gene is called significant. For group 1 we chose all 15 microarrays from all high-calorie-fed mice (HIGHCAL) and for group 2 we chose all 8 CR microarrays. We included gene duplicates and controls in the SAM procedure but removed them after, leaving a non-redundant set of 8,347 genes.

For GenAge associations we used only mouse genes and mouse orthologs of human genes listed in GenAge. For human genes we conservatively used only mouse orthologs that share the same function and assigned Entrez gene name listed in the Homologene database (release 59). For GO classification we used the web tool FuncAssociate [35]. FuncAssociate uses a Monte Carlo simulation approach and returns all overrepresented GO classes for a given set of input genes relative to the gene universe from which this subset is derived, along with several descriptive statistics, including *P*-value based on Fisher's exact test, and adjusted *P*-value based on 1000 null hypothesis simulations to correct for simultaneous testing of multiple non-independent hypotheses. We analyzed six different sets of genes using FuncAssociate: upregulated, downregulated, and all genes, from both permissive and restrictive data sets. FuncAssociate analyses were run using the unordered input option. The input gene universe for FuncAssociate consisted of all genes on our microarrays, which FuncAssociate culled down to a final set of 17,851 unique genes.

### Western blots and quantitative RT–PCR

For Western blots mouse livers (100 mg) were homogenized in 1× NuPAGE LDS loading buffer (cat. #NP0007, Invitrogen, Carlsbad, CA) with 1× protease inhibitor mix (cat. #8340, Sigma-Aldrich, St. Louis, MO), 1× Phosphatase Inhibitor Cocktail Set 1 (cat. #524624, Calbiochem, San Diego, CA) and 1× Phosphatase Inhibitor Cocktail Set 2 (cat. #524625, Calbiochem, San Diego, CA). The homogenates were sonicated for 15 seconds to reduce viscosity, and centrifuged at 15,000×g for 10 minutes to remove insoluble material. Samples were subjected to standard SDS-PAGE and probed by Western blot using phospho-specific antibodies against phospho-4E-BP1 (Thr70) (cat. #9455, Cell Signaling Technology, Danvers, MA), phospho-p70 S6 kinase (Thr389) (cat. #9205, Cell Signaling Technology, Danvers, MA) and phospho-p70 S6 kinase (Thr421/Ser424) (cat. # 9204, Cell Signaling Technology, Danvers, MA). Membranes were stripped using Restore Western Blot Stripping Buffer (cat. #21059, Pierce Biotechnology, Inc., Rockford, IL) for 30 minutes at 37°C, and were reprobed with antibodies directed against total p70 S6 kinase (cat. #9202, Cell Signaling Technology, Danvers, MA) or total 4E-BP1 (cat. #9452, Cell Signaling Technology, Danvers, MA). Membrane bound HRP-conjugated secondary antibody was detected using SuperSignal West Femto Maximum Sensitivity Substrate (cat. #34095, Pierce Biotechnology, Inc., Rockford, IL). Quantitation of protein bands was performed using Quantity One software (Bio-Rad Laboratories, Inc., Hercules, CA).

We used quantitative RT-PCR (Q-RT-PCR) to verify microarray results for specific genes of interest. A listing of primers used in Q-RT-PCR assays can be found in **Table S4** and all fold-changes can be found in **Table S1**. Two control transcripts (*Masp2 and D3Ucla1*) were chosen; of all transcripts with high but non-saturable expression levels, they were among the transcripts with the lowest variance across all tested regimens. Primers that amplify a predicted amplicon of at least 100 bases and less than 250 bases were favored over others. All of the primers for Q-RT-PCR were designed to span exon junctions so as to avoid amplification of any potential contaminating genomic DNA. Nevertheless, in addition to “no RNA” negative control reactions for each primer, for all RNAs we performed “no RT” PCR reactions (i.e., without reverse transcriptase) in order to ensure that there is no contaminating genomic DNA that would result in amplification products in the Q-RT-PCRs. First strand cDNA synthesis was performed with 10 µg of total RNA, random hexamers, and SuperScriptIII Reverse Transcriptase (Invitrogen).

Each quantitative PCR reaction was carried out in iCycler IQ Real-Time Detection Systems (Bio-Rad), in a total volume of 25 µl, which contained 1× iQ SyBr Green Supermix (BioRad) and 200 nM forward and reverse primers. PCR amplification for each cDNA sample was performed in at least triplicate wells. The quantitative PCR conditions were as follows: 3 min at 95°C, followed by a total of 45 three-temperature cycles (30 sec at 95°C, 30 sec at between 60.5°C and 65.0°C, and 45 sec at 72°C). Relative gene expression data analysis was carried out with the standard curve method [69], [70], and statistical significance of fold-changes was calculated using a t-test, requiring a *P*-value of at least 0.05 for significance [71], [72].

## Supporting Information

Table S1 Quantitative RT-PCR fold-changes and array fold-changes for selected transcripts. Positive fold changes are ratios of CR/HIGHCAL and negative fold changes are negative values of ratios of HIGHCAL/CR. Unless denoted as not significant (NS) all listed RT-PCR values are significant at P<0.05.
(0.02 MB XLS)

Table S2 All genes significantly changed in CR relative to HIGHCAL. Fold changes are provided by Significance Analysis of Microarrays (SAM) software, and are approximate values averaged over all CRmax/HIGHCAL conditions. Negative fold changes are represented as the negative inverse (-HIGHCAL/CRmax). Stat Score (d) is the modified t-statistic returned by SAM. FC Rank is the ranking of genes in descending order from largest to smallest fold change and separate rankings are provided for upregulated and downregulated genes. Separate statistical rankings are provided for upregulated and downregulated genes and are combined into a single list. q-value is given as a percent from SAM and the restrictive set of genes comprises those with a q-value<1.0%.
(0.75 MB XLS)

Table S3 Significantly over-represented Gene Ontology (GO) terms returned by FuncAssociate at an adjusted P-value cutoff of p<0.05. GO terms are listed in descending order of significance for each input gene set. Six sets are shown: genes UPREGULATED by CR, genes DOWNREGULATED by CR, and the combined list of genes both up- and DOWNREGULATED by CR, for both statistical cutoffs, q<0.01 and q<0.1. Rank is the position in the attribute list ranked by significance of association with the given query; N is the number of genes in the query with this attribute; X is the number of genes overall in the query universe with this attribute; LOD is the natural log of the odds ratio; P is the single hypothesis one-sided P-value of the association between attribute and query (based on Fisher's Exact Test); and P-adj is the adjusted P-value: fraction (as a %) of 1000 null-hypothesis simulations having attributes with this single-hypothesis P value or smaller.
(0.10 MB XLS)

Table S4 Primer sequences used for Q-RT-PCR.
(0.02 MB XLS)
